# Efficacy and Safety of Percutaneous Nephrolithotomy in Children: A Single-Center Retrospective Analysis

**DOI:** 10.7759/cureus.109189

**Published:** 2026-05-19

**Authors:** Madan Gopal Bhardwaj, Lalit Kumar, Yashasvi Singh, Sameer Trivedi, Ujwal Kumar, Narayan Shubham, Tej Bali Singh

**Affiliations:** 1 Urology, Institute of Medical Sciences, Banaras Hindu University, Varanasi, IND; 2 General Surgery, Pandit Bhagwat Dayal Sharma Post Graduate Institute of Medical Sciences, Rohtak, IND; 3 Preventive Medicine, Institute of Medical Sciences, Banaras Hindu University, Varanasi, IND

**Keywords:** metabolic lithogenesis, mini-pcnl, pediatric pcnl, pediatric urolithiasis, percutaneous nephrolithotomy

## Abstract

Introduction: Pediatric urolithiasis is increasing globally, with metabolic and environmental factors contributing to recurrence despite advances in minimally invasive surgery. Percutaneous nephrolithotomy (PCNL) is now the preferred treatment for large or complex stones in children, yet long-term data on metabolic predictors of recurrence remain limited.

Objective: This study aims to evaluate the safety, efficacy, and recurrence predictors following PCNL in pediatric patients at a tertiary center in northern India.

Study design: A retrospective analysis was conducted on 27 children (≤14 years) who underwent PCNL between January 2021 and May 2023. Preoperative demographic, biochemical, and radiological parameters were recorded. Stone burden was assessed using CT urography or NCCT (non-contrast computed tomography) and graded by Guy’s Stone Score (GSS). Standard or mini-PCNL techniques were selected based on stone characteristics. Postoperative biochemical changes, stone composition, outcomes, and recurrence over two years were evaluated.

Results: The mean age was 8.8 ± 3.4 years (range 1-14), with a male-to-female ratio of 1.7:1. GSS Grade 2 stones predominated (59.3%), with a mean size of 1.9 ± 0.6 cm. Complete clearance was achieved in all patients, with a mean hospital stay of 3 ± 1 days. Hemoglobin decreased by 0.81 g/dL (p = 0.016), while creatinine remained unchanged. No major complications occurred; minor Clavien-Dindo I-II events were seen in 44.4%. Calcium oxalate stones accounted for 66.7%. At a mean follow-up of 37.5 months, recurrence occurred in 22.2%, mainly in children with acidic urine and low specific gravity. Multivariate analysis revealed no independent predictors, although urine pH and specific gravity showed predictive trends.

Conclusion: PCNL demonstrated favorable stone clearance with acceptable morbidity in this cohort. Recurrence appeared to be influenced by hydration and metabolic factors, although conclusions are exploratory due to the small sample size.

## Introduction

India reported the highest incidence, mortality, and DALYs (disability-adjusted life years) of pediatric stone disease in 2021, with 1,064,248 cases, 9 deaths, and 3,638 DALYs [1-3]. Northern and northwestern Indian states (Punjab, Haryana, Rajasthan, and Uttar Pradesh) show the highest prevalence, linked to low protein intake, high-oxalate diets, and recurrent infections [4,5]. Infection-related ammonium urate and struvite stones, once dominant, are yielding to calcium-based calculi driven by metabolic abnormalities and lifestyle changes [6,7]. Metabolic causes (hypercalciuria, hypocitraturia, cystinuria, and hyperoxaluria) are now identified in 30-60% of pediatric cases [8,9]. In affluent regions, obesity and insulin resistance promote hyperuricosuria and reduced urinary pH, further driving stone risk [10,11]. School-aged children and adolescents more often show calcium-based calculi linked to diet and lifestyle [12]. Male predominance is consistent worldwide, although infection-related regions show reduced gender disparity [13].

Recurrence rates are alarmingly high, up to 50% within five years if metabolic abnormalities remain untreated [14]. The chronic nature of the disease brings psychosocial strain, school absenteeism, and repeated medical therapies and surgical interventions (retrograde intrarenal surgery, percutaneous nephrolithotomy (PCNL), and extracorporeal shockwave lithotripsy) [15,16]. We hypothesized that PCNL is a highly efficacious and hemodynamically safe modality for managing pediatric renal stones and that long-term stone recurrence is driven primarily by metabolic rather than surgical factors. To test this, we conducted a retrospective study at a tertiary care center in Uttar Pradesh in pediatric patients undergoing PCNL. Our primary objective was to evaluate the short-term safety and efficacy of the procedure. Our secondary objective was to explore long-term metabolic, biochemical, and clinical associations with stone recurrence over a two-year follow-up period.

## Materials and methods

Study setting

This retrospective study was conducted from January 2021 to May 2023 and included 27 pediatric patients aged 0-14 years at a tertiary care center located in northern India. The institutional ethics committee at Banaras Hindu University approved the study via letter no. IMS/IEC/2024/7311. Informed consent for surgery was obtained from the children’s parents or legal guardians at the time of admission, and ethical approval was obtained for retrospective analysis.

Study population

This study included a consecutive series of children aged ≤14 years with renal stones who underwent PCNL at our center. Our institution serves as a major tertiary referral center, capturing a diverse patient population from local and neighboring districts with varying socioeconomic and dietary backgrounds. Exclusion criteria included age >14 years, incomplete data, and patients lost to follow-up.

Data collection

Data were retrieved from the hospital database. Baseline parameters, including age, hemoglobin, creatinine, serum PTH, vitamin D, calcium, phosphate, and uric acid, were recorded. CT urography or NCCT (non-contrast computed tomography) kidney-urinary bladder, depending on the patient’s creatinine, was used to calculate the stone burden, which was then classified according to Guy’s Stone Score (GSS). For consistency, stone size and GSS were calculated using the non-contrast phase of imaging. Due to the practical challenges of obtaining accurate 24-hour urine collections in young, non-toilet-trained, or uncooperative children, baseline metabolic evaluation primarily relied on spot urinalysis (pH, specific gravity) and serum biochemistry.

PCNL using the bull’s-eye technique was performed under general anesthesia in the prone position using either a standard PCNL or a mini-PCNL approach, depending on stone size, location, availability and functioning of surgical equipment, and patient anatomy. In standard PCNL, renal access was achieved under fluoroscopic guidance using an 18-G needle, followed by tract dilation up to 24-30 Fr with Amplatz or metallic dilators, and stone fragmentation was performed using pneumatic lithotripsy or vaporized using a holmium or thulium fiber laser.

The mini-PCNL technique employed similar access under fluoroscopic guidance. Tract dilation was limited to 16 Fr, allowing the use of mini nephroscopes (12 Fr) and low-pressure irrigation to minimize parenchymal trauma and bleeding. Stone clearance was achieved through active fragmentation and suction, and all fragments were retrieved under direct vision. In some cases, a laser was used to vaporize the stones. A nephrostomy tube was placed at the surgeon’s discretion based on intraoperative findings and hemostasis. The DJ (double-J) stent was usually removed at 6 weeks. The choice between mini- and standard PCNL was individualized to balance maximal stone clearance with minimal morbidity, particularly in younger children.

Postoperative assessment

Postoperative parameters, including hemoglobin and serum creatinine levels, were recorded. The chemical composition of retrieved stones was analyzed using Fourier-transform infrared spectroscopy (FTIR). The follow-up imaging modality (USG (ultrasonography), X-ray of the kidneys, ureters, and bladder (KUB), or NCCT) was tailored to the individual, adhering to the ALARA (As Low As Reasonably Achievable) principle to minimize radiation exposure in the pediatric cohort. Ultrasound was primarily used as the first-line follow-up modality. Follow-up assessments were conducted at two weeks, six weeks, three months, one year, and two years postoperatively to evaluate recurrence.

Outcome measures

Stone clearance rate, recurrence rate, and the modality used for treating recurrence were recorded.

Statistical analysis

Data were analyzed using IBM SPSS Statistics for Windows, Version 26 (Released 2019; IBM Corp., Armonk, New York). Continuous variables were presented as mean ± SD or median (IQR) based on the Shapiro-Wilk test. Categorical data were presented as counts and percentages. Pre- and postoperative biochemical values were compared using paired t-tests or Wilcoxon tests. Associations between categorical variables were assessed using chi-square or Fisher’s exact tests. Factors with p < 0.1 in univariate analysis were entered into an exploratory multivariate logistic regression model to assess potential associations with recurrence. Given the small cohort size and low number of events, this analysis was treated as exploratory and hypothesis-generating rather than strictly predictive.

## Results

Baseline characteristics and demographics

A total of 27 pediatric patients underwent PCNL during the study period. Standard PCNL was performed in 18 cases and mini-PCNL in 9. One patient, aged 1 year, underwent bilateral sequential mini-PCNL at an interval of 6 weeks. On metabolic evaluation, he was found to have a 100% uric acid stone and was advised prophylactic alkalizer therapy for 3 months.

The mean age of the cohort was 8.81 ± 3.36 years (range: 1-14 years). The majority were aged 9-12 years (n = 17, 62.9%), followed by 5-8 years (n = 7, 25.9%), under 5 years (n = 2, 7.4%), and those aged 12 years (n = 1, 3.7%) of the cohort. The average hemoglobin level was 11.09 ± 1.38 g/dL, and 44.4% of the children had levels between 9.6 and 11.0 g/dL, indicating that nearly half of the group had mild preoperative anemia.

The mean serum creatinine was 0.69 ± 0.34 mg/dL, with 81.5% of cases within the 0.4-0.89 mg/dL range, indicating preserved renal function. The mean blood urea was 32.52 ± 24.20 mg/dL, with most values between 21 and 30 mg/dL. Electrolyte evaluation showed that serum calcium levels were largely within the 8.6-9.5 mg/dL range (mean 8.89 ± 0.84 mg/dL), and the mean serum phosphate was 4.15 ± 0.81 mg/dL. Serum uric acid ranged chiefly between 2.0 and 4.5 mg/dL. Vitamin D levels showed insufficiency (<30 ng/mL) in 37% of cases.

Urinalysis demonstrated acidic urine (mean pH <5) in 70.4% (n = 19) and normal specific gravity (1.010-1.024) in 77.7% (n = 21). The mean stone size was approximately 1.9 cm, with one-third (n = 9, 33.3%) measuring ≤1.4 cm and another 25.9% (n = 7) between 1.5 and 1.9 cm. According to the GSS, most cases were of moderate complexity, Grade 2 (n = 16, 59.3%), followed by Grade 1 (n = 8, 29.6%), while Grades 3-4 accounted for 11.1% (n = 3). The baseline characteristics are presented in Table 1. 

**Table 1:** Baseline characteristics Age, hemoglobin, creatinine, urea, serum calcium, phosphate, uric acid, Vitamin D, and stone size have been represented in mean ± SD. Age groups, gender ratios, GSS grades, and stone compositions have been described in percentages. Hb: hemoglobin; Cr: creatinine; SG = specific gravity; GSS = Guy’s Stone Score; Ca-ox = calcium oxalate.

Parameter	Value	Range/Percentage
Age (years)	8.81 ± 3.36 (n=27)	1–14 (range)
Age groups (years)	1–4 (n=2)	7.4%
	5–8 (n=7)	25.9%
	9–12 (n=17)	62.9%
	>12 (n=1)	3.7%
	Total	100%
Sex	Male, n=17	63.0%
	Female, n=10	37.0%
Hemoglobin (g/dL)	11.09 ± 1.38	8.2–13.8 (range)
Serum creatinine (mg/dL)	0.69 ± 0.34	0.4–2.1 (range)
Blood urea (mg/dL)	32.52 ± 24.20	10.8–128 (range)
Serum calcium (mg/dL)	8.89 ± 0.84	8.6–9.5 (range)
Serum phosphate (mg/dL)	4.15 ± 0.81	2.0–5.4(range)
Serum uric acid (mg/dL)	3.7 ± 1.0	2.3–5.5 (range)
Vitamin D (ng/mL)	37.9 ± 18.5	15–76 (range)
Stone size (cm)	1.9 ± 0.6	0.9–3.1 (range)
Guy’s Stone Score	Grade 1 (n=8)	29.6%
	Grade 2 (n=16)	59.3%
	Grades 3–4 (n=3)	11.1%
Stone composition	Calcium oxalate (n=18)	66.7%
	Uric acid (n=9)	33.3%

Postoperative outcomes and follow-up

The average hemoglobin level after surgery was 10.28 ± 1.66 g/dL, and 30.8% of the children had levels between 10.0 and 10.9 g/dL. There was only a modest decline of about 0.8 g/dL from baseline. The mean postoperative serum creatinine was 0.68 ± 0.27 mg/dL, remaining comparable to preoperative values, suggesting preserved renal function after PCNL (Table 2). There were no residual calculi on follow-up imaging, and 100% of cases achieved complete stone clearance. The mean hospital stay was 3 ± 1 days. 

**Table 2:** Change in haemoglobin and creatinine after PCNL Data are presented as mean ± SD. p-value is considered statistically significant at <0.05. Hb: hemoglobin; Cr: creatinine; SD: standard deviation; Δ: difference (post-pre); PCNL: percutaneous nephrolithotomy; Wilcoxon: Wilcoxon signed-rank test represented by z value.

Parameter	Pre-op Mean ± SD	Post-op Mean ± SD	Mean Δ (Post–Pre)	Test Statistic (Wilcoxon)	p-value
Hemoglobin (g/dL)	11.09 ± 1.38	10.28 ± 1.66	−0.81	Z=-2.41	0.016
Creatinine (mg/dL)	0.70 ± 0.34	0.69 ± 0.27	−0.01	Z= - 0.22	0.826

No significant intraoperative or postoperative complications were observed. Minor events such as transient fever or mild hematuria occurred in a few cases and resolved with conservative management. All postoperative complications were graded according to the Clavien-Dindo classification, with only Grade I-II events observed in 12 patients (I 75%, II 25%), including fever, pain at the surgical site, and hematuria not requiring transfusion. No Grade ≥III complications were reported.

Stone analysis showed that calcium oxalate was the most common type (n = 18, 66.7%), while uric acid stones accounted for 33.3% (n = 9). A subanalysis of calcium oxalate stones revealed a mean monohydrate content of 21.22 ± 6.06% and a dihydrate component of 78.78 ± 6.06%, indicating a predominance of the dihydrate form in most cases.

Patients were followed for a minimum of two years and beyond. The mean follow-up duration was 37.5 ± 7.25 months (23-52). At one year, all patients (100%) were asymptomatic and had normal kidney function. At two years, six patients (22.2%) developed stone recurrence on imaging (USG, X-ray KUB, or CT), while the remaining 21 (77.8%) remained stone-free. No significant complications or renal impairment were reported during follow-up. Of the six patients with recurrence, three underwent successful ESWL, two were managed with medical therapy, and one underwent PCNL.

The 22.2% recurrence rate observed at two years is likely multifactorial, primarily driven by metabolic and hydration-related factors rather than surgical or anatomical causes. Patients with recurrence showed lower urine specific gravity and consistently lower urinary pH. These conditions may favor calcium oxalate crystallization and suggest ongoing metabolic derangements such as hyperoxaluria or hypocitraturia. Vitamin D insufficiency in a substantial subset may further contribute to altered calcium metabolism and recurrent lithogenesis. Overall, recurrence in this cohort appears metabolic in origin, highlighting the importance of postoperative hydration optimization and metabolic follow-up in pediatric stone prevention.

Univariate analysis of recurrence

Patients with recurrence (n = 6) tended to have lower urine specific gravity (mean 1.016 ± 0.006 vs 1.021 ± 0.005; p = 0.074). They also showed slightly higher hemoglobin levels and lower urine pH, although these differences were not statistically significant (p > 0.1). No differences were observed in age, creatinine, calcium, phosphate, uric acid, vitamin D, or GSS. Stone composition showed no independent correlation with recurrence (p = 0.35) (Table 3).

**Table 3:** Univariate analysis results Age, hemoglobin, creatinine, urea, serum calcium, phosphate, uric acid, urine specific gravity, Vitamin D, and stone size are represented as mean ± SD. Age groups, gender ratios, GSS grades, and stone compositions are presented as percentages. p-value is considered statistically significant at <0.05. *Note on GSS: Statistical test statistics (Fisher's exact/Kruskal-Wallis H) for GSS are reported for methodological transparency; however, due to the highly constrained sample size of complex stones (GSS 3–4, n=3), these comparative metrics are severely underpowered and should be interpreted strictly descriptively. H denotes Kruskal–Wallis test statistic; U denotes Mann–Whitney U test statistic. Hb: hemoglobin; Cr: creatinine; Ca: calcium; P: phosphate; Vit D: vitamin D; SG: specific gravity; GSS: Guy’s Stone Score; Ca-ox: calcium oxalate; OR: odds ratio.

Variable	Recurrence (n=6)	No Recurrence (n=21)	Test Statistic (U or Fisher)	p-value
Age (years)	8.9±3.2	8.7±3.4	U = 60.5	0.88
Hemoglobin (g/dL)	11.4±1.1	10.9±1.3	U=43.0	0.21
Serum creatinine (mg/dL)	0.71±0.30	0.68±0.35	U=58.5	0.79
Serum calcium (mg/dL)	8.95±0.82	8.88±0.86	U=60.0	0.74
Serum phosphate (mg/dL)	4.21±0.77	4.13±0.83	U=59.0	0.69
Serum uric acid (mg/dL)	3.8±1.1	3.6±0.9	U=54.5	0.61
Vitamin D (ng/mL)	34.2±16.8	39.1±18.9	U=51.0	0.43
Urine pH	4.9±0.3	5.2±0.4	U = 35.0	0.12
Specific gravity	1.016±0.006	1.021±0.005	U = 31.0	0.074
Stone size (cm)	2.0±0.5	1.8±0.6	U = 46.5	0.32
GSS ≥ 3*	2 (33.3%)	1 (4.7%)	Fisher's exact	0.545
Stone size ≥ 2 cm	2 (25.0%)	5(75.0%)	Fisher's exact	1.000
Calcium oxalate	4 (66.7%)	14 (66.7%)	X^2^ = 0.00	0.367

Exploratory analysis of recurrence associations

Variables with univariate p < 0.10 (specific gravity) and clinically relevant covariates (urine pH) were entered into a logistic regression model. Table 4 describes the results of the multivariate analysis. Despite the constraints of a small sample size, the model yielded an AUC of 0.73 and a pseudo-R² of 0.136. Because the model is heavily constrained by the low number of recurrence events (n = 6), the resulting AUC and pseudo-R² are reported strictly for descriptive transparency and cannot be interpreted as stable predictive metrics. 

**Table 4:** Multivariate analysis results OR: odds ratio; AUC: area under the curve.

Variable	Adjusted OR	95% CI	p-value
Urine specific gravity	0.41	0.11-1.61	0.203
Urine pH	0.49	0.15-1.62	0.241
Model performance	AUC: 0.73	Pseudo-R^2^: 0.136	

Although no single biochemical parameter independently predicted recurrence, the observed pattern of lower specific gravity and acidic urine in recurrent cases suggests that chronic dehydration and metabolic acid load may be contributing factors in pediatric lithogenesis. The predominance of dihydrate in calcium oxalate stones likely facilitated efficient lithotripsy clearance and the 100% initial stone-free rate, underscoring the safety and efficacy of PCNL in this population. When stratified by stone features, no statistically significant association was observed between recurrence and stone composition (p = 0.367). Due to the extremely small number of patients with highly complex GSS 3-4 stones (n = 3), formal comparative testing was not performed for stone complexity, although descriptively, recurrence occurred in one of the three complex cases. 

The mean perioperative hemoglobin decline was 0.81 ± 0.72 g/dL, consistent with the minimal decline typically observed in pediatric PCNL. When stratified by stone size, GSS, and stone composition, no statistically significant differences were observed (p > 0.05 for all comparisons). Descriptively, children with larger stones (≥2 cm) and higher GSS (3-4) demonstrated a numerically greater mean hemoglobin reduction; however, due to the small subgroup size of GSS 3-4 (n = 3), statistical comparisons are unreliable. Similarly, patients with calcium oxalate stones had a slightly greater mean fall in hemoglobin than those with uric acid calculi, but this difference was not statistically significant (p = 0.365). The change in serum creatinine (ΔCr) was negligible across all strata, averaging −0.01 ± 0.09 mg/dL, indicating stable postoperative renal function regardless of stone size, complexity, or composition. No correlation was found between GSS and ΔCr (p = 0.437) or between stone composition and ΔCr (p = 0.935). These results affirm that PCNL in pediatric patients is associated with minimal hemoglobin decline and preserved renal function, even in complex or large calculi, provided meticulous surgical technique and hemostasis are maintained (Table 5).

**Table 5:** Correlation of postoperative fall of haemoglobin and creatinine with clinical parameters Statistical significance was assessed using the Kruskal-Wallis or Mann-Whitney U test, with p-values considered significant at <0.05. H denotes Kruskal–Wallis test statistic; U denotes Mann–Whitney U test statistic. p-value is considered statistically significant at <0.05. *Note on Guy's Stone Score (GSS): Statistical test statistics (Fisher's exact/Kruskal-Wallis H) for GSS are reported for methodological transparency; however, due to the highly constrained sample size of complex stones (GSS 3–4, n=3), these comparative metrics are severely underpowered and should be interpreted strictly descriptively. Δ: change (post-pre); Hb: hemoglobin; Cr: creatinine; GSS: Guy’s Stone Score; Ca-ox: calcium oxalate; ns: not significant.

Parameter	Compared Using	Test Statistic (H or U)	p-value
Δ Hb (g/dL)	GSS*	H = 1.54	0.461
	Composition	U = 65.5	0.365
Δ Cr (mg/dL)	GSS*	H = 1.65	0.437
	Composition	U = 81.5	0.935

Although patients with larger stones (≥2 cm) and higher GSS (≥3) demonstrated numerically higher recurrence, the differences were not statistically significant owing to small subgroup sizes. Calcium oxalate and uric acid stones showed similar recurrence patterns, reinforcing that metabolic and hydration factors (e.g., specific gravity, urine pH) were more critical determinants of recurrence than anatomical complexity or composition. These findings suggest that postoperative metabolic optimization and hydration remain essential even after complete surgical clearance (Table 6). 

**Table 6:** Correlation of recurrence with stone morphology and composition Statistical significance was assessed using the chi-square or Fisher’s exact test. p-value is considered statistically significant at <0.05. GSS: Guy’s Stone Score; Ca-ox: calcium oxalate; χ²: chi-square test; ns: not significant; PCNL: percutaneous nephrolithotomy.

Parameter	Category Compared	Recurrence Rate (%)	p-value	Test Used
Stone size	<2 cm vs ≥2.0 cm	20.0 vs 25.0	1.0	Fisher
Guy’s Stone Score	1–2 vs 3–4	20.8 vs 33.3	0.545	Fisher
Stone composition	Calcium oxalate vs uric acid	22.2 vs 22.2	1.0	χ²

## Discussion

Our study reinforces that PCNL is a safe treatment modality for pediatric urolithiasis, achieving an immediate 100% stone-free rate with minimal morbidity. The mean age of 8.8 years and male predominance in our cohort align with previously published pediatric series from India, Turkey, and the Middle East, where climatic and dietary factors amplify lithogenic risk [1-3]. The regional predominance of calcium oxalate calculi (66.7%) over infection-related stones mirrors the global epidemiologic transition from infectious to metabolic urolithiasis in children. Complete clearance was achieved even in children with moderate-to-complex stones (GSS 2-3), suggesting that PCNL can be used as a definitive procedure for high stone burden and complex calculi in pediatric kidneys. Comparable clearance rates and low transfusion rates have been reported by Kapoor et al. (83.9% clearance; 90.3% with dual therapy) [17,18]. The mean hemoglobin drop of 0.8 g/dL and the absence of Grade ≥III Clavien-Dindo complications further affirm procedural safety.

Both standard (n = 18) and mini-PCNL (n = 9) were successfully utilized in our cohort to achieve stone clearance, with the choice of approach tailored to patient anatomy, surgeon discretion, and equipment availability. While our sample size and unstratified design preclude a methodologically valid comparison of efficacy or safety between the two access techniques, the descriptive success of both approaches aligns with established clinical standards. The smaller tract size (14-18 Fr) utilized in mini-PCNL is well documented to minimize parenchymal trauma, reduce bleeding, and obviate the need for prolonged drainage [19]. Recent multicenter analyses advocate its use even in infants, provided fluoroscopic exposure and irrigation pressures are optimized. A recent meta-analysis in children evaluating tubeless PCNL reported shorter operative time and hospital stay, with improved complication and pain profiles [20]. When compared with retrograde intrarenal surgery (RIRS), mini-PCNL demonstrated superior stone-free rates and lower postoperative fever and complication rates, although operative time was longer and heterogeneity was high [21]. A meta-analysis comparing PCNL to ureteroscopy for stones larger than 15 mm showed stone clearance rates of 94.0% for PCNL and 55.0% for ureteroscopy, with significantly lower pain intensity and urinary symptoms one week after surgery [22].

The recurrence rate of 22.2% observed at 2 years aligns with long-term pediatric series, in which recurrence ranges from 15% to 50% depending on metabolic surveillance and compliance with preventive measures [23]. In our cohort, recurrence did not demonstrate statistically significant associations with stone size, composition, surgical complexity, or baseline biochemical parameters. Recurrent cases numerically exhibited lower urine specific gravity and persistently acidic urine; although these parameters did not reach statistical significance due to the small sample size, this descriptive trend may favor calcium oxalate crystallization and suggests ongoing metabolic derangements. Rather than drawing definitive causal inferences, these observations generate a clinical hypothesis that aligns with findings by Milliner and Stapleton [24], who emphasized that inadequate hydration and metabolic acidosis perpetuate calcium oxalate crystallization even after surgical clearance. Vitamin D insufficiency in one-third of our cohort may also influence calcium-phosphate balance and predispose to recurrence, as described by Sas et al. [25].

Stone composition analysis revealed a predominance of calcium oxalate dihydrate, a relatively softer variant associated with favorable lithotripsy response and shorter operative times. This compositional bias may have contributed to the high stone-free rates and absence of residual fragments [26]. While uric acid stones constituted one-third of cases, recurrence patterns were similar between calcium oxalate and uric acid groups, suggesting that intrinsic metabolic correction and hydration monitoring outweigh compositional differences in preventing recurrence.

Renal function remained preserved postoperatively, as reflected by stable creatinine levels and absence of renal impairment on follow-up. These outcomes are consistent with those reported by Ganpule et al., who demonstrated minimal scarring and stable glomerular filtration rate (GFR) following PCNL. Even in higher GSS grades and in the standard PCNL group, no notable rise in creatinine or major complications was observed, affirming the safety of PCNL when performed by experienced surgeons under controlled irrigation pressure [27].

Collectively, our data suggest that pediatric PCNL is both efficacious and safe. The modest recurrence rate highlights the importance of close follow-up, structured metabolic evaluation, and parental counseling to maintain adequate fluid intake, dietary moderation, and periodic biochemical monitoring. As the pediatric stone disease paradigm evolves from surgical correction to metabolic control, the surgeon’s role must extend beyond stone clearance to long-term prevention.

Limitations

This study has several critical limitations that must be acknowledged. First, the retrospective design and small sample size (n = 27) mean the study is significantly underpowered. Specifically, our multivariate logistic regression is limited by a low number of recurrence events (n = 6), which violates the statistical rule of events per variable. Consequently, this regression model is strictly exploratory and hypothesis-generating, rather than genuinely predictive.

Second, there is considerable heterogeneity within our cohort. We pooled patients across a wide age range (1-14 years) and utilized different surgical techniques (standard and mini-PCNL) without a formalized comparator group or sufficient sample size to perform age-stratified subgroup analyses. Because the choice of standard versus mini-PCNL was based on surgeon discretion and equipment availability, these observational comparisons are subject to selection bias. Importantly, only three patients in our cohort presented with high-complexity GSS 3-4 stones. Consequently, statistical comparisons involving GSS are heavily underpowered and inherently unreliable; outcomes related to GSS 3-4 are reported strictly descriptively and should not be overemphasized.

Third, our reported 100% immediate stone-free rate must be interpreted with caution; this outcome likely reflects selection bias and the relatively moderate mean stone size (1.9 cm) of our cohort and may lack broad external validity when applied to centers managing massive or complex pediatric staghorn calculi.

Fourth, due to logistical constraints and the inherent difficulty of 24-hour urine collection in young children, the metabolic work-up was limited primarily to spot urinalysis and serum biochemistry. Finally, follow-up imaging was heterogeneous (USG, X-ray, and CT) to comply with pediatric ALARA (As Low As Reasonably Achievable) radiation principles, which introduces a potential detection bias for small, asymptomatic recurrences. Despite these limitations, the uniformity of our surgical approach and the explicit focus on the transition from surgical clearance to metabolic prevention highlight important clinical trends that warrant future, adequately powered prospective studies.

## Conclusions

In our specific cohort, which predominantly featured low-to-moderate complexity stones, PCNL offered excellent immediate stone clearance with minimal morbidity and sustained renal safety. No significant complications (Grade ≥III) were encountered. At the two-year follow-up, recurrence was observed in 22.2% of patients. While our study was not adequately powered to establish definitive statistical predictors, our exploratory data suggest that these recurrences may be associated with underlying metabolic and hydration factors rather than surgical shortcomings. Despite the limitations of our retrospective design and incomplete metabolic panels, these clinical observations highlight the potential value of postoperative hydration optimization, dietary counseling, and close biochemical surveillance. Ultimately, pediatric stone management must be viewed not just as a surgical event but as a continuum of care that integrates procedural clearance with long-term metabolic stewardship, a paradigm that warrants validation in larger, adequately powered prospective trials.
